# Accelerated neuronal aging *in vitro* ∼melting watch ∼

**DOI:** 10.3389/fnagi.2022.868770

**Published:** 2022-08-09

**Authors:** Emi Inagaki, Sho Yoshimatsu, Hideyuki Okano

**Affiliations:** ^1^Department of Physiology, Keio University School of Medicine, Tokyo, Japan; ^2^Department of Ophthalmology, Keio University School of Medicine, Tokyo, Japan; ^3^Japanese Society for the Promotion of Science (JSPS), Tokyo, Japan

**Keywords:** iPSCs (induced pluripotent stem cells), disease modeling, aging, human model, *in vitro* model

## Abstract

In developed countries, the aging of the population and the associated increase in age-related diseases are causing major unresolved medical, social, and environmental matters. Therefore, research on aging has become one of the most important and urgent issues in life sciences. If the molecular mechanisms of the onset and progression of neurodegenerative diseases are elucidated, we can expect to develop disease-modifying methods to prevent neurodegeneration itself. Since the discovery of induced pluripotent stem cells (iPSCs), there has been an explosion of disease models using disease-specific iPSCs derived from patient-derived somatic cells. By inducing the differentiation of iPSCs into neurons, disease models that reflect the patient-derived pathology can be reproduced in culture dishes, and are playing an active role in elucidating new pathological mechanisms and as a platform for new drug discovery. At the same time, however, we are faced with a new problem: how to recapitulate aging in culture dishes. It has been pointed out that cells differentiated from pluripotent stem cells are juvenile, retain embryonic traits, and may not be fully mature. Therefore, attempts are being made to induce cell maturation, senescence, and stress signals through culture conditions. It has also been reported that direct conversion of fibroblasts into neurons can reproduce human neurons with an aged phenotype. Here, we outline some state-of-the-art insights into models of neuronal aging *in vitro*. New frontiers in which stem cells and methods for inducing differentiation of tissue regeneration can be applied to aging research are just now approaching, and we need to keep a close eye on them. These models are forefront and intended to advance our knowledge of the molecular mechanisms of aging and contribute to the development of novel therapies for human neurodegenerative diseases associated with aging.

## Introduction: The need for an *in vitro* neuronal aging platform

The global aging society has prompted a new era to overcome aging-associated diseases in basic research fields. Since the WHO proposed “healthy life expectancy” in 2000, there has been a growing interest in living longer and healthier lives in today’s super-aging society, especially in developed countries. The trend toward rapid aging is particularly relevant to the Sustainable Development Goals (SDGs), which relate to eradicating poverty as well as ensuring healthy lives and wellbeing at all ages (United Nations, 2015). As the economic burden of age-related health problems increases, effective ways to control them are being explored. The importance of taking a bird’s eye view of the various domains and attempting to link and integrate them across the board has also been pointed out. Thus, the problem of elucidating the mechanisms and treatment of human aging and age-related diseases has entered a new phase, reflecting the imminent aging of society.

Aging is associated with physical deterioration and increases the risk of developing various diseases and death (Harman, 1981). Although the mechanism of aging is still unclear, it is partially clear today that this aging process has done with certain regulatory mechanisms. Aging is associated with various factors including genomic instability, telomere shortening, cellular senescence, epigenetic changes, loss of protein homeostasis, alterations in nutrient signaling, mitochondrial dysfunction, stem cell depletion, and alterations in cell–cell interactions, which manifest and promote aging traits (López-Otín et al., 2013). The aging process is accompanied by alterations in the molecular basis of aging, which in turn leads to the breakdown of homeostatic mechanisms throughout the body and accelerates the aging process by reducing tissue plasticity and regenerative capacity. This is because that aging is a universal process characterized by the accumulation of biological changes (Haigis and Yankner, 2010; López-Otín et al., 2013; Mattson and Arumugam, 2018). Approaches for mechanistic elucidation of the onset and progression in various neurodegenerative diseases, including Alzheimer’s disease, Parkinson’s disease, and amyotrophic lateral sclerosis (ALS) have led to the development of disease-modifying therapies to prevent neurodegeneration itself (Kritsilis et al., 2018; Hou et al., 2019; Bobkova et al., 2020; Gendron et al., 2020). There are several potential candidates for disease-modifying therapies for ALS, AD, and PD, including AAV-mediated gene therapies and antibody-based approaches and cell transplantation (Doi et al., 2020; Amado and Davidson, 2021; Tampi et al., 2021; Cummings et al., 2022; Rahimpour et al., 2022; Shi et al., 2022; Takahashi and Mashima, 2022). However, these approaches are yet under clinical trials or debates, and not thoroughly validated in human.

Humans are extremely long-lived among primates. Conventional aging research mainly attributes to a decline in the force of natural selection with time and encompasses many kinds of model organisms including yeasts, worms, flies, and rodents. It has been suggested that there are evolutionary and fundamental mechanistic differences between these models and humans. Therefore, it seems that new and exciting challenges in aging research should focus on human-specific aging. Considering ways to fully understand human aging can contribute to a new sustainable society aimed at longevity and health (Johnson, 2015).

Takahashi and Yamanaka (2006) and Takahashi et al. (2007) reported a technology to produce induced pluripotent stem cells (iPSCs) from somatic fibroblasts of mice and humans. Since then, disease-specific iPSCs established from patients’ somatic cells have been expected to be applied as a differentiation model (Pang et al., 2011). Pathological analysis of neurodegenerative disease models using iPSCs derived from patients with neurodegenerative diseases has been reported, thus iPSCs have become a powerful research tool (Liu et al., 2012; Imaizumi and Okano, 2014, 2021; Okano and Yamanaka, 2014). iPSC models have the potential to elucidate pathological conditions and develop new therapies. Patient-derived iPSCs are cells that have been rejuvenated while retaining the genetic background of the patient involved in the disease. By selectively inducing differentiation of these cells into cells that are damaged and degenerated by the disease, the cells may serve as an *in vitro* model for reproducing the disease process *in vivo*. The majority of successful cases of pathological modeling with iPSCs to date have been of the inherited, early-onset-type diseases (Seibler et al., 2011; Imaizumi et al., 2012; Soldner and Jaenisch, 2012; Pomp and Colman, 2013; Engle et al., 2018; Doss and Sachinidis, 2019; Chang et al., 2020; D’Souza et al., 2021).

Nevertheless, there are still various problems in the analysis using disease modeling with iPSCs. The first problem is its juvenility compared to the *in vivo* tissue. In other words, iPSC-derived cells *in vitro* can only reproduce a limited period of development, seemingly the embryonic or early postnatal period, when compared to the developmental timeline of a human individual, and may not exhibit an adult phenotype (Mariani et al., 2012; Nicholas et al., 2013). Therefore, it is essential to develop a culture method that promotes maturation and senescence in order to reliably and reproducibly capture the phenotype of neuronal senescence in adults (Liu et al., 2012). The next problem is that it is very difficult to induce age-dependent changes in culture. Strategies that have been implemented to induce cellular senescence include overexpression of *Progerin*, shortening of telomeres using chemical compounds, and the addition of toxic stress (Miller et al., 2013; Dong et al., 2014; Vera et al., 2016). However, aging is a very complex process that is very difficult to mimic in a culture system, it is controversial whether any of these strategies are sufficient to reproduce the complexity of senescence itself *in vitro*. Although these strategies have contributed to latent aspects of the disease phenotype, it is unclear whether they have resulted in age-dependent changes or not. Thus, new fundamental technologies are expected to be developed that will cause a paradigm shift in aging and regeneration.

Here, in this paper, we review the latest research on various innovative methods to promote neuronal maturation, aging *in vitro.* If, in the future, anyone can reproduce functionally mature cells of the appropriate age, this could not only be an important milestone for the understanding of the pathogenesis of late-onset neurodegenerative diseases and thus assist to identify a druggable target, but also contribute to elucidating the big challenge in life science, such as the principle of aging. We introduce an overview of the major papers reported so far, and discuss what we should tackle toward the super aging society.

## Things to keep in consideration neuronal aging with induced pluripotent stem cell

In current disease modeling approaches using iPSCs, researchers are trying to rewind time from somatic cells to their pre-birth state (to an approximately peri-implantation epiblast stage), and from there to reproduce the aging process to some extent in a culture dish. We would like to outline what is rejuvenated during the establishment of iPSCs from somatic cells by summarizing previous reports. Early embryos, especially those at the one-cell to morula stage, have the potential to give rise to any types of cells and can divide indefinitely, but as development progresses and cell division is repeated, they differentiate into cells with finite lifespans and specific functions. These specific differentiations are inherently irreversible. However, the technology to rejuvenate somatic cells against this irreversible flow is the technology to create iPSCs.

### Cellular rejuvenation: Erasing age-dependent changes

In the iPSC reprogramming technology, overexpression of Yamanaka factors [*Oct4*, *Sox2*, *Klf4*, and *c-Myc* (so-called OSKM)] can transform terminally differentiated cells into a pluripotent state (Takahashi and Yamanaka, 2006; Malik and Rao, 2013) (Table 1). Among the four transcription factors, *Oct4* and *Sox2* activate genes necessary for maintaining cell pluripotency, while *c-Myc* and *Klf4* are known to regulate cell proliferation. Cooperative binding of transcription factors orchestrates reprogramming (Jaenisch and Young, 2008; Soufi et al., 2012; Chronis et al., 2017). Then, iPSC reprogramming is carried out through the global remodeling of epigenetic marks and many of the epigenetic marks are remodeled during the iPSC reprogramming process, including DNA methylation, histones’ post-translational modifications, and topological chromatin remodeling (Doi et al., 2009; Lister et al., 2011; Hansson and Chien, 2012; Hansson et al., 2012; Buganim et al., 2013; Takahashi and Yamanaka, 2016). iPSC reprogramming resets the epigenetic alternations that somatic cells have acquired through development and aging, making the body clock similar to that of embryonic stem cells (Rando and Chang, 2012; Horvath, 2013; Kerepesi et al., 2021). In the process, molecular traces associated with aging and maturation are partially or fully erased during the process of pluripotency induction. In other words, age-related epigenomic changes, such as the DNA methylation state that human tissues acquire over time, can be restored by reprogramming (Manukyan and Singh, 2012). These phenomena are difficult to control during aging (Singh and Newman, 2018).

**TABLE 1:** Summary of iN attempts in previous studies (review and mechanistical analysis papers were excluded).

References	Cell	iN induction factors	Summary of results
Vierbuchen et al. (2010)	Mouse fibroblasts	*Ascl1, Myt1l, Brn2*	∼19.5% Tuj-1 positive cells
Ambasudhan et al. (2011)	Human fibroblasts	miR-124, *MYT1L, BRN2*	∼11.2% Tuj-1 positive cells
Pfisterer et al. (2011)	Human fibroblasts	*ASCL1, MYT1L, BRN2, LMX1A, FOXA2*	∼20% Tuj-1 positive cells
			∼1% TH positive cells
Yoo et al. (2011)	Human fibroblasts	miR-9/9* and miR-124	∼5% Tuj-1 positive cells (only miRs)
		*NEUROD2, ASCL1, MYT1L*	∼80% Tuj-1 positive cells (NEUROD2/ASCL1/MYT1L + miRs)
Liu et al. (2013)	Human lung fibroblasts	*NEUROG2*	∼90% Tuj-1 positive cells (mostly cholinergic)
Pereira et al. (2014)	Human fibroblasts	*ASCL1, MYT1L, BRN2* with SMAD inhibitors	∼42% Tuj-1 positive cells
Li et al. (2015)	Mouse fibroblasts	Forskolin, ISX9, CHIR99021, SB431542, I-BET151	∼90% Tuj-1 positive cells

Tuj-1, a pan-neuronal marker; TH, a dopaminergic, adrenergic, and noradrenergic neuronal marker.

Previous studies have shown that iPSC reprogramming not only makes cells pluripotent, but also rejuvenates a variety of age-related features such as nuclear morphology and composition, nuclear membrane composition, heterochromatin content, DNA damage accumulation, and telomere length (Mahmoudi and Brunet, 2012). Global cellular characteristics such as aging, proliferation, mitochondrial metabolism, and accumulation of oxidative stress are also rejuvenated (Liu et al., 2012). It has been suggested to have a tendency to differentiate with a bias toward somatic epigenetic memory and the ability to form teratomas (Kim et al., 2011; Buganim et al., 2013). The mechanism by which reprogramming rejuvenates the cell fate decision is still unclear, but the developmental resetting will be a unique detriment to the further use of iPSCs as a research material for late-onset disease modeling.

### Juvenility

Methods for inducing differentiation of human iPSCs (hiPSCs) into a variety of cell lineages have been rapidly developed, and reproducible and robust protocols have been established in various areas. Despite these early successes, it has become apparent that differentiated cells from iPSCs resemble those of early embryos rather than those of adult tissues (Spence et al., 2011; Patterson et al., 2012; Nicholas et al., 2013; Abdelalim and Emara, 2015; Batalov and Feinberg, 2015). The same situation was observed in the differences in transcriptional and metabolic mechanisms, which have been noted in hepatocyte-like cells derived from iPSCs compared to adult primary hepatocytes (Baxter et al., 2015). Recent global gene expression and network analyses have also demonstrated this trend, suggesting the embryonic stage of iPSC-derived cells. Moreover, gene networks associated with maturation and aging are suppressed in familial and sporadic ALS disease strains (Lister et al., 2011; Ho et al., 2016). These contribute to the fact that while the gene expression profiles associated with the induction of differentiation in early embryos are still being elucidated, the details of induction associated with late development, including postnatal development, are still unresolved in each tissue (Bellin et al., 2012; Mertens et al., 2018).

To solve this problem, several attempts have been performed by manipulating neuronal inducing factor(s). A previous report has shown that dual inhibition of SMAD signaling promotes induction of neural differentiation (Chambers et al., 2009). In an attempt to generate homogeneous neurons, Zhang et al. (2016) succeeded in differentiating pluripotent stem cells into neurons with an almost 100% yield in 2 weeks by overexpressing the transcription factor *Neurogenin-2* (*NEUROG2*), a bHLH-type proneuronal factor (Liu et al., 2013). This strategy follows the same theory used to induce cell initiation, which is to reset the entire biological system by inducing overexpression of a small number of transcription factors. Recently, microRNA-9/9* (miR-9/9*) and microRNA-124 (miR-124) had been implicated in efficient neuronal differentiation and functional maturity (Sun et al., 2013; Lu and Yoo, 2018). More recently, we have devised an induction method by combining *NEUROG2* and miR-9/9*-124 overexpression that made iPSCs exhibit neuronal disease phenotypes rapidly with a higher maturity (Ishikawa et al., 2020). Another attempt was made by optimizing the culture media, for example, the inhibition of Notch activity by the gamma-secretase inhibitor N-[N-(3,5-difluorophenacetyl)-L-alanyl]-S-phenylglycine t-butyl ester (DAPT) was used to modulate the neuronal differentiation. It enabled marked acceleration of differentiation, moreover, DAPT-mediated Notch inhibition delayed G1/S-phase transition (Borghese et al., 2010). However, the addition of DAPT itself may negatively affect the recapitulation of the amyloid and Tau pathology, so not seemingly being suitable for promoting the disease phenotype of AD (Raja et al., 2016). The details of differentiation methods have been summarized in several review articles (Engle et al., 2018; Giacomelli et al., 2022).

Another strategies in *in vitro* cell culture techniques increasingly seek to recapitulate complex tissue- and organ-level phenotypes, such as spheroid-based organoids, three-dimensional (3D) cell culture systems, and even microfluidic devices (Lancaster and Knoblich, 2014; Camp et al., 2015; Blokzijl et al., 2016; Choi et al., 2016; Qian et al., 2016; Hu et al., 2018; Cho et al., 2021; Giandomenico et al., 2021; Holloway et al., 2021; Luo and Li, 2021). Each method offers its own advantages: spheroid can partially recreate the 3D structure of physiological brain tissue but presents challenges in controlling the size and composition in the culture. 3D scaffold cultures promote the recapitulation of the intravital cellular microenvironment, but at the expense of physiological relevance to other aspects. Microfluidic cultures may be able to add different cell types, promote microenvironment, and enhance translatability. It has also been reported that long-term culture of organoids for 10 months confirms complex mechanisms such as spatiotemporally complex brain network activity as seen in the EEG of preterm infants. Reports of long-term cultures of these organoids are mimicking some of the key components of the complex developmental programs inherent in living organisms (Hu et al., 2018; Trujillo et al., 2019; Gordon et al., 2021). However, there is a concern about the ethical aspects of human brain organoid research based on the official ISSCR (the International Society for Stem Cell Research) statements (Sawai et al., 2019; Ide et al., 2021; Lovell-Badge et al., 2021). But iPSC-based studies can help rapid-growing health care problems, for example, the cellular basis of Maternal Zika Virus infection was analyzed using an *in vitro* infection model of iPSCs-derived organoids (Muffat et al., 2018). For more information, please refer to the following reviews by respective experts (Passier et al., 2016; Parr et al., 2017; Castelli et al., 2019; Seto and Eiraku, 2019; Chiaradia and Lancaster, 2020; Ooi et al., 2020; Fares et al., 2021a,b; Holloway et al., 2021; Josephine Boder and Banerjee, 2021; Luo and Li, 2021; Qian and Tcw, 2021).

## Strategies for manipulating the cell age of induced pluripotent stem cells-derived induced cells

### Manifesting stress phenotype by chemical compound

Exposing cells to stressing environments may contribute to the development of specific diseases, such as oxidative stress. This notion has been exploited to accelerate the emergence of pathological phenotypes *in vitro*. Addition of Hydrogen peroxide (H_2_O_2_), reactive oxygen species (ROS), radiation, and other stimuli on cells increases DNA damage and endogenous ROS, inhibits neurite outgrowth, and causes the aggregation of specific proteins and nucleotides (Seibler et al., 2011; Dong et al., 2014). So, when disease-specific iPSC-derived neurons exhibit almost no evident phenotype, it seems a better way to add chemical stressors for promoting disease phenotypes (Nguyen et al., 2011; Imaizumi et al., 2012). There are many known phenotypes that stress-induced cellular senescence. For more details, please refer to several reviews, but this is done *in vitro* with drugs and small molecule compounds to mimic stress in the microenvironment. Therefore, it should be noted that this is a different perspective from aging (Haigis and Yankner, 2010; Petrova et al., 2016; Noren Hooten and Evans, 2017; Hernandez-Segura et al., 2018).

### Accelerated neuronal aging: Overexpression of *Progerin*

In this section, we will discuss the attempt to challenge the recapitulation of aging phenotypes *in vitro*. Miller et al. (2013) developed an *in vitro* preparation that is useful for measuring the extent to which transient expression of Progerin is associated with this “aging” molecular profile. A very unique study to induce aging using iPSCs was the first to examine the expression of early aging-related protein variants in iPSC-derived induced neural cells (Brennand, 2013; Miller et al., 2013). The causative gene of Hutchinson-Gilford Progeria Syndrome (HGPS), a form of premature aging syndrome, is known to be *Lamin A* (*LMNA*), which encodes a nuclear membrane component protein. LMNA and Lamin C are two types of A-type lamins. LMNA and Lamin C are produced by selective splicing after the transcription of the *LAMA* gene. LMNA interacts with nuclear chromatin and is involved in nuclear degradation and remodeling during cell division, as well as telomere dynamics. The cause of HGPS is a mutation in the *LAMA* gene, called *Progerin*. Progerin production is thought to induce structural and functional changes in the nuclear membrane, leading to premature aging. Ectopic expression of *Progerin* causes DNA damage, telomere shortening, p53-dependent changes in gene expression regulation, and induction of cellular senescence and cell death. Also, the expression of *Progerin* can be used to induce an increase in aging indicators such as γH2AX foci, ROS accumulation, and a loss of H3K9me3 in neurons. More recently, Coyne et al. (2020) showed that ALS-related nuclear pathology including nuclear envelope dysfunctions can be reproduced in an optimized condition of motor neuron induction for accelerating neuronal maturation without the overexpression of *Progerin*. Therefore, respective disease-specific mechanisms may also trigger the recapitulation of neuropathology *in vitro*.

### Shortening telomere length

Another way to induce aging is to make changes to telomeres, one of the hallmarks of aging. It is well-known that telomeres shorten with aging due to the principal mechanism in eukaryotic genomic DNA replication, except cells in early-stage embryos and germ cells expressing *Telomerase* (*TERT*), which encodes an enzyme that causes telomere elongation (Allsopp et al., 1995; Greider, 1996; Hug and Lingner, 2006; Rocca et al., 2019). Telomerase activity and telomere length have been suggested to directly affect the tissue regenerative capacity and aging of stem cells (Blasco, 2007). Telomere length measurement is increasingly recognized as a clinical gauge for age-related disease risk (Mather et al., 2011; Fasching, 2018). There are still many aspects of the effects and mechanisms of telomere shortening that remain largely unknown. Rejuvenation of telomeres with various lengths has been found in iPSCs. Mechanisms of telomere length regulation during induction and proliferation of iPSCs remain elusive, although iPSCs show the *TERT* expression similar to that of ESCs (Ly, 2011; Wang et al., 2012; Le et al., 2014; Fu et al., 2018). Several aspects of telomere biology may be responsible for altered telomere dynamics in iPSCs, and unique reports have focused on these phenomena. The chemical compound BIBR1532 was discovered as a potent and selective telomerase inhibitor capable of inducing senescence in human cancer cells (Pascolo et al., 2002). In 2016, Vera et al. (2016) found BIBR1532 could shorten the telomeres of iPSCs-derived neurons. They confirmed aging-related traits such as generation of ROS and DNA damage in neurons with shortened telomeres. However, BIBR1532 compatible with continued iPSC culture was proved as insufficient to induce detectable telomerase inhibition, which resulted in unsuccessful recapitulation of age-related phenotypes in their conditions (Pandya et al., 2021). Thus, these effects are unclear in the models of neurodegenerative diseases, and the full impact of this treatment on post-mitotic cells remains to be systematically scrutinized.

### Neuropathological phenotypes that are currently challenging to model

**FIGURE 1:**
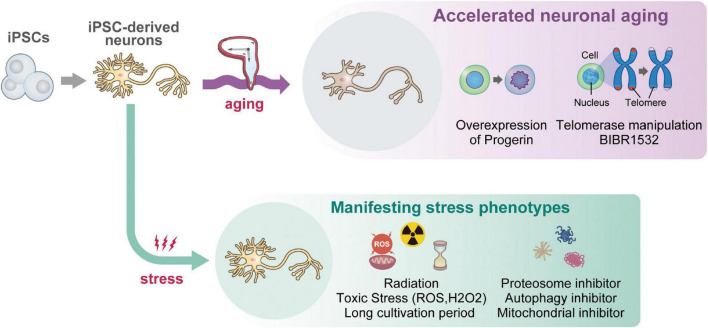
A graphical schematic of current iPSC-based approaches for reproducing “aging” *in vitro*. Starting from iPSCs (once rejuvenated), because iPSC-derived neurons themselves show juvenile phenotypes, multiple approaches including overexpression of Progerin, telomere manipulation, and supplementation of stress molecules were attempted so far. Although a recent report succeeded in reproducing age-related nuclear pathology of ALS (Coyne et al., 2020), most attempts were unsuccessful for recapitulating neuropathological features (i.e., neurofibrillary tangle), age-related DNA damage response and methylation, and mitochondrial aging features, which are listed as “Currently infeasible phenotypes” *in vitro*.

Although various disease phenotypes have been recapitulated in culture dishes, neuropathological phenotypes have mainly been analyzed with the qualified and very limited resources of human autopsy brains animal disease models. Impaired proteostasis of proteins has been noted as a feature associated with aging and neurodegenerative processes (Daniele et al., 2018). Protein degradation is mainly regulated by both the ubiquitin-proteasome and autophagy-lysosome systems (Komatsu et al., 2007; Kurtishi et al., 2019). Age-related reduction of the ubiquitin proteasome system in the aging brain promotes the accumulation of protein aggregates (Klaips et al., 2018). It has been suggested that the aggregation of abnormal proteins in the brain is one of the mechanisms that induce the neurotoxicity observed in neurodegenerative diseases (Daniele et al., 2018), such as the neurofibrillary tangle caused by hyperphosphorylated tau aggregation in AD brains (Masters et al., 1985; Perry et al., 1987), the formation of Lewy bodies in PD brains (Litvan et al., 1998), or Skein-like inclusions or Busina bodies in ALS (Leigh et al., 1991; Okamoto et al., 2008). Previous research showed evidence that partially replicated the early-stage disease’s pathophysiology by utilization of iPSCs derived neuronal cells *in vitro* (Sances et al., 2016; Cobb et al., 2018; Chang et al., 2020; Klimmt et al., 2020; Baena-Montes et al., 2021; Fares et al., 2021a,b; Giacomelli et al., 2022). If late-stage pathophysiology with such findings can be reproduced in culture dishes, the scope of research may be expanded to find ways to reduce or reverse neurodegenerative pathology by manipulating new therapeutic pathways (Figure 1).

## Transdifferentiation (iNs; direct converted neuronal cells from dermal fibroblasts)

At present, the neurons that reliably reflect age-dependent changes are iNs derived from dermal fibroblasts by transdifferentiation. Direct reprogramming, the induction of neuronal cells from fibroblasts, was first reported in 2010. By using lentiviruses to introduce three factors, Brn2, Ascl1, and Myt1l, selected from 19 transcription factors that act specifically on neuronal cells, they succeeded in inducing neuronal cells (iN cells). The key to this reprogramming is the pioneer factor Ascl1, although the overexpression of *Ascl1* alone induced iN cells with a relatively low efficiency (Vierbuchen et al., 2010). In addition, the importance of *Ascl1* in epigenomic variation and transcription factor binding during iN cell induction has been suggested (Wapinski et al., 2013). Various research groups have developed efficient iN methods by introducing defined transcription factors including *ASCL1*, miRNAs, or short hairpin RNAs (shRNAs), resulting in the direct change of the cell fate (Pfisterer et al., 2011; Yoo et al., 2011; Lu and Yoo, 2018). It has been reported that small molecule cocktails could also promote iN production (Liu et al., 2013; Pereira et al., 2014; Li et al., 2015).

Notable works in these studies include overexpression of miR-9/9* and miR124, which are specifically expressed in neurons, and multiple transcription factors have been combinatorially used (Yoo et al., 2011; Sun et al., 2013; Ishikawa et al., 2020; Nemoto et al., 2020). Since epigenetic remodeling has been suggested to be involved in the acquisition of pluripotency, it is believed that multiple transcription factors and epigenetic regulation are required to truly implement cell fate transformation. miR-9/9* and miR124 have been reported to induce dramatic chromatin reconfiguration and topologically open neuronal subtype specific loci (Abernathy and Yoo, 2015; Takahashi and Yamanaka, 2016; Abernathy et al., 2017). Some targets for neuronal maturation have been reported (Lu et al., 2021). It is reported that iN cells from Alzheimer’s disease patients exhibited impaired neuronal maturation (Mertens et al., 2021). Researchers also succeeded in establishing protocols for the generation of several disease-relevant neuronal subtypes (Liu and Yoo, 2019; Cates et al., 2021; Church et al., 2021).

In recent years, the nuclear pore complex and nucleocytoplasmic communication have received much attention as factors and targets of protein changes in aging. Interestingly, a decrease in the expression of *RANBP17*, one of the nucleocytoplasmic transport factors observed in aging, has been reported to maintain this age-dependent trait as it has been observed in iNs (Mertens et al., 2015). It has been found that the epigenome in neurons induced by this method retains the same age-related traits as that of postmortem brain samples. Further identification of a cocktail of factors that will faithfully convert fibroblasts into mature, functioning neurons comparable to those *in vivo* is a critical challenge.

One of the major problems to be overcome with iN is the limited number of cells that can be obtained: in contrast to the inexhaustible cell resource of iPSCs. The original resource of iNs is fibroblasts, and their expandability is usually limited to 20–30 passages mainly due to the telomere shortening by mitosis without *TERT* expression. This makes it somewhat of a hindrance when hundreds of millions of cells are needed for experiments such as high-throughput screening. Other concerns include the efficiency of induction and whether there are any intermediates that can clonally proliferate during iN conversion. We hope that new technological innovations will solve these problems in the future.

Alternatively, attempts for directly deriving multipotent neural stem cells/neural progenitors from somatic fibroblasts have been made (Kim et al., 2011; Han et al., 2012; Ring et al., 2012; Lu et al., 2013; Yoshimatsu et al., 2021). However, these cells, so-called induced neural stem cell/neural progenitors (iNSC/iNPs), tend to acquire a more posterior (caudate) fate along with expansion, therefore this feature is not favorable for obtaining neurons with an anterior fate, such as cortical excitatory and inhibitory neurons.

**FIGURE 2:**
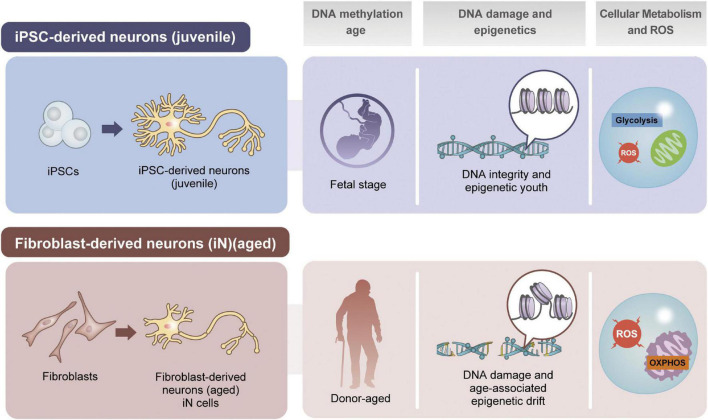
Comparison of iPSC- or fibroblast-based approaches for disease recapitulation. In many aspects such as genomic DNA methylation, damage and epigenetic factors, and cellular metabolic status are different in neurons depending on the resources of induction.

In addition, the rejuvenation effect by the direct reprogramming into iNSC/iNPs has not been thoroughly investigated, it is possible that aged features are partially erased during reprogramming. Regarding this issue, aging marks are not generally investigated in respective studies owing to the solid definition of “cellular aging” is not fully established. Thus, this consensus is necessary in cell biology and neuroscience research fields (Figure 2).

Furthermore, other primary cells, i.e., white blood cells and urine-derived cells have been used for directly deriving neurons through reprogramming (Zhang et al., 2016; Tanabe et al., 2018; Xu et al., 2019). Since the collection of blood or urine is clinically general and less invasive than skin biopsy, these cells can be a good resource once robust and highly reproducible iN reprogramming methods are established. Preliminarily, we have collected blood and urine from patients with neurological diseases for elucidating the neuropathology through iN reprogramming (unpublished). Furthermore, we are assessing the aged status of the iN cells in multimodal aspects. We believe these approaches would also help enhance the robustness of neurological disease research.

## Insights from related fields

Since neurodegeneration and aging are closely related, a variety of factors have been reported so far (Castelli et al., 2019; Bobkova et al., 2020). In this section, we would like to give an overall perspective of some of the areas of particular interest in the related fields. Several master genes involved in neural differentiation and brain aging have been identified. In the past, neuron-restrictive silencer factor/repressor element 1 silencing transcription factor (NRSF/(REST) was originally identified as a transcription factor that represses several neuron-specific genes in non-neuronal cell types. In addition to its repressive action, REST also functions in sympathetic nervous system cells during the neurite outgrowth phase of early neurogenesis. The expression of REST is negligible in young and mature neurons, but high in aged neurons, indicating that REST is responsible for the overall regulation of age-dependent neuronal changes (Lu et al., 2014). This factor protects neurons from oxidative stress and also from amyloid β (Aβ) aggregation stress, which is a major problem in AD. In the elderly, the level of *REST* expression in the brain is high in healthy aging individuals, but it tends to be clearly lower in AD patients. In other words, REST seems to be a master regulator of neurogenesis as well as a regulator of the aging brain (Lunyak and Rosenfeld, 2005). Recently, Meyer et al. (2019) from the Yanker’s group, generated iPSCs from cells of sporadic AD patients and healthy controls, and comprehensively analyzed gene expression in these iPSC-derived neural cells. These results suggest that it is possible to influence aging by increasing the expression level of *REST* and decreasing the activity of excitatory neurons (Meyer et al., 2019). *REST* expression also disappeared from nucleus of neurons in PD, another leading neurodegenerative disease (Kawamura et al., 2019). In patients with Huntington’s disease, *Huntingtin* (*HTT*) is mutated. The mutant HTT protein loses its ability to bind to REST, which results in translocation of REST into the nucleus, and suppresses the expression of neural-related genes, including *Brain derived neurotrophic factor* (*BDNF*) by binding to neuron-restrictive silencer element (NRSE) (Zuccato et al., 2003). Furthermore, with regard to REST, nuclease-sensitive regions in nucleasomes, so-called liberated regions, have been identified in both hiPSCs and mouse embryonic stem cells (mESCs). In mESC, it has been found that there are many binding sites for REST and CCCTC-binding factor (CTCF) in the liberated regions. Thus, it is becoming clear that REST is a transcription factor of interest as a target for neuronal aging.

In recent years, accumulating evidence has emerged that reduced nucleo-cytoplasmic transport (NCT) and damage to the nuclear membrane and nuclear pores are associated with neurodegenerative diseases and physiological aging (Pujol et al., 2002; D’Angelo et al., 2009; Freibaum et al., 2015; Rubin and Taatjes, 2015; von Appen et al., 2015; Woerner et al., 2016; Schlachetzki et al., 2020; Coyne and Rothstein, 2022). Basically, Alterations in NCT regulation have also been reported as one of the hallmarks associated with aging (Broers et al., 2006; Scaffidi and Misteli, 2006). It has also been suggested that the NCT capacity of specific transport pathways or substrates (nuclear or extra-nuclear transport) changes with cellular senescence (Wente and Rout, 2010). NCTs are a class of proteins with nuclear localization signals and nuclear export signals, respectively. The amount of importing α and β family members, which are NCT factors, decreases with aging. As mentioned above, Progerin is known to accumulate in normal aging, and in HGPS, a diseased form of premature aging, abnormal nuclear membrane morphology occurs. It is also reported that the amount of Ras-related nuclear protein (Ran), a low molecular weight G protein that plays an important role in active nuclear-cytoplasmic transport, is decreased in the nucleus of fibroblasts from HGPS patients, and the nuclear-cytoplasmic concentration gradient is disrupted (Kelley et al., 2011). NCTs are known to become leaky with aging (D’Angelo et al., 2009). In neurodegenerative diseases, there is growing evidence to suggest that NCT changes contribute to pathophysiology, particularly in ALS. The differences in disease-related conditions may also be related to the fact that nucleocytoplasmic transport proteins and nucleoporins are specific cell types. Various reports on neurodegenerative diseases suggest that age-related decline in overall nuclear integrity, including nuclei and NCTs, may contribute to the development of neurodegenerative diseases. We refer to several reviews (Bitetto and Di Fonzo, 2020; Coyne and Rothstein, 2022).

Loss or dysfunction of mitochondrial function in brain aging and age-related diseases has been noted in many studies and is one of the key aging-related phenotypic features (Klaips et al., 2018; Mattson and Arumugam, 2018). Since mature neurons are non-proliferative cells, age-related changes can accumulate *in vivo* and eventually cause loss of mitochondrial function. Aging has been shown to impair a variety of mitochondrial functions, including mitochondrial dynamics, transport, mitophagy, and energy homeostasis (Canto et al., 2015; Panchal and Tiwari, 2019; Cabral-Costa and Kowaltowski, 2020; Messina et al., 2020). One important class of proteins governing the effect is the Sirtuin family, which interacts with other aging-related accumulable proteins. Sirtuins are an evolutionally conserved family of Nicotinamide Adenine Dinucleotide (NAD) histone deacetylases and play a critical part in aging and longevity control in diverse model organisms including yeasts, worms, flies, mice, and humans (Imai et al., 2000; Satoh et al., 2017; Jardim et al., 2018). NAD is a pivotal metabolite involved in cellular bioenergetics, genomic stability, mitochondrial homeostasis, dynamics, adaptive stress responses, and cell survival (Li and Sauve, 2015). Moreover, multiple NAD-dependent enzymes are involved in synaptic plasticity and neuronal stress resistance. NAD homeostasis appears to be of paramount importance to health span and longevity, and its dysregulation is associated with neurodegenerative diseases (Katsyuba and Auwerx, 2017; Lautrup et al., 2019). Complementary strategies to NAD, such as the use of anti-CD38 antibody inhibitors to reduce NAD consumption, or the intermediate precursors of NAD such as NMN (nicotinamide mononucleotide) or NR (nicotinamide riboside) have been taken to translational research worldwide (Martens et al., 2018; Yoshino et al., 2018, 2021; Irie et al., 2020; Reiten et al., 2021; Brakedal et al., 2022).

## Conclusion

As outlined in this paper, various facts have been revealed by a variety of innovative technologies in recent years, but the different pathological phenotypes of the elderly are very complex, mainly caused by various factors that accumulate over a biological time span in human beings. Therefore, elucidating the molecular basis of age-related neurodegenerative diseases should be a major challenge for future life science research. Since it is impossible to directly observe live human brain at a cellular level, iPSCs are undoubtedly a very attractive research material due to the unlimited expansion potential that is advantageous for high-throughput drug screening. However, studies using iPSCs face a new problem in that the accumulation of age-dependent changes in a dish is necessary to reproduce the disease phenotypes, especially late-onset ones, although the cells were once rejuvenated by iPSC reprogramming. There may be a trade-off between rejuvenation and aging induction, but there is a need to synergize differentiation and aging induction to create a new platform for *in vitro* research on human aging. On the other hand, iN technology may overcome the reprogramming-associated rejuvenating effects, however, it also suffers from its fundamental problem such as limited expansion of the original cells. Therefore, we infer iN can be applied to personalized medicine which only requires a small number of cells. We believe that better understanding of the properties and limitations of iPSCs and iNs would lead to future research breakthrough(s). Understanding these concepts, limitations, and expectations will allow us to maximize the potential of these technologies to understand and treat neurodegenerative diseases and even intervene in the aging process itself.

## Author contributions

El was involved in drafting the original manuscript and revision. YS substantially contributed to the revision of the manuscript. HO was involved in the critical revision of the manuscript. All authors have approved the submitted version of the manuscript.
